# How People's Motivational System and Situational Motivation Influence Their Risky Financial Choices

**DOI:** 10.3389/fpsyg.2016.01360

**Published:** 2016-08-31

**Authors:** Katarzyna Sekścińska, Dominika Agnieszka Maison, Agata Trzcińska

**Affiliations:** ^1^Faculty of Psychology, University of WarsawWarsaw, Poland; ^2^Institute of Social Science, University of WarsawWarsaw, Poland

**Keywords:** chronic motivation system, situational promotion vs. prevention motivation, risk preferences, financial choices, regulatory focus theory

## Abstract

People's preferences for risks have been a subject of interest to researchers in both the economy and psychology fields over the last few years. This has given rise to many important findings about the role of psychological factors that influence people's choices. The presented studies focused on the role of motivational systems (described by Higgins in the Regulatory Focus Theory) in explaining people's financial choices. The main goal was to examine the relationship between people's chronic promotion and prevention motivational system and their propensity to (1) invest, (2) undertake investment risks, and (3) assume financial risks in gambling tasks in both the gain and loss decision-making frame. Moreover, we aimed to investigate how chronic motivational systems confronted with situationally induced promotion and prevention motivation would affect people's propensity to invest and embrace financial risks. Two CAWI studies on a Polish national representative sample (*N*_**1**_ = 1093; *N*_**2**_ = 1096) were conducted. The second study consisted of two waves with a 2-week break. The studies provided evidence of higher chronic promotion motivation as well as higher prevention motivation associated with the propensity to invest; however, induced promotion motivation results in a lower propensity to invest compared to induced prevention motivation. Participants with an activated promotion system built more risky portfolios than individuals with an induced prevention system. Moreover, participants with a low chronic promotion system built more risky portfolios than individuals with a high promotion motivation system as long as their prevention system was also low. In terms of gambling decisions in both the gain and loss frame, a higher level of chronic promotion motivation and situationally induced promotion motivation were related to the preference for the non-sure option over the sure one.

## Introduction

In traditional economic theories, a decision maker is described as a fully rational individual whose choices are the result of estimating the relevant probabilities and outcomes. Moreover, a decision maker is focused on maximizing expected value or utility (Von Neumann and Morgenstern, 2004). This way of thinking about decision makers has already been modified, largely due to the prospect theory (Kahneman and Tversky, 1979). Nowadays, it is well known that people's financial decisions not only depend on the relevant probability and outcomes, but also on many other external factors (such as different decision domains and frames) that are subjectively important when deciding, as well as internal factors, such as personality traits, attitudes, motives, or needs.

### Risk preferences in different domains and frames

To date, many researchers have investigated the structure of people's risk preferences. They focused on different decision domains like the financial, social, or health domains. The findings showed that, contrary to classical decision theories, risky decisions are affected by various factors like framing (e.g., Tversky and Kahneman, 1981), the source of the probability information (Hertwig et al., 2004), any previous experience (e.g., Sekścińska, 2015a), the decision maker's individual traits (e.g., Campbell et al., 2004), or even the activation of a different social role (Sekścińska et al., 2016). This signifies that decisions made in different domains (even when they are made concurrently) should not be described by the same and stable utility function. Slovic (1972) showed that people may be risk-averse in one domain and risk-seeking in another because the variance of returns is not a consistent predictor of risk-taking. Other researchers (Weber et al., 2002; Hanoch et al., 2006) have also showed that people are not consistently risk-averse or risk-seeking across all decision domains. Furthermore, Kusev et al. (2009) provided evidence that variation in the content of decisions leads to variation in financial risk preferences. Moreover, Vlaev et al. (2010) postulated that risk preferences may differ depending on the decision domain because the reference point may be different for various domains, for instance, monetary gambling vs. insurance. Vlaev et al. (2010) conducted a study in which they distinguished between monetary gambles, hazard losses, investment, insurance, pension provision, job salary change, and mortgage buying. The results of the study showed that risk attitude estimation is strongly dependent on the financial decision domain.

Kahneman and Tversky (1979) in their Prospect theory showed the function of value for gains and losses. They argued that this function is: (1) defined on deviations from the reference point; (2) concave for gains and convex for losses; and (3) steeper for losses than for gains (ibid.). These arguments lead to the conclusion that people may behave differently depending on the frame of the decision—gains or losses. The findings from studies presented by the authors of the Prospect theory (Kahneman and Tversky, 1979, 2000) followed by studies conducted by Kusev et al. (2009, 2011) and Vlaev et al. (2010), support the hypothesis that decisions under risk may vary depending on the frame of the decision: framed as either loss or gain.

Taking the findings from the aforementioned studies into account, it seems worthwhile investigating the mechanisms underlying risky financial decisions in order to distinguish between the propensity to make the decision in different domains (i.e., investment and gambling) and in different frames (i.e., gain vs. loss).

### Promotion and prevention focuses as motivational systems and situational motivation

The factors that determine people's preferences for risks have been a subject of interest to researchers in the field of psychology for the last decades. Many findings have identified different psychological variables that influence risky financial decisions, for example, personality traits (e.g., narcissism—Foster et al., 2009, 2011; Sekścińska, 2015b; sensation-seeking and locus of control—Wong and Carducci, 2016), attitudes toward money (e.g., Sekścińska, 2015b), and affect-based motivation (e.g., Aspara and Tikkanen, 2011). In this paper, we would like to focus on one psychological factor that seems to be important in explaining people's financial risk preferences based on a theoretical framework as well as on a few studies conducted to date in this field. The focus will be on the promotion and prevention motivational system described by Higgins in the Regulatory Focus Theory (Higgins, 1997, 1998).

The Regulatory Focus Theory refers to the two distinct motivational systems that regulate all goal-directed behaviors (Higgins, 1997, 1998). Higgins (1997) distinguishes between promotion and prevention motivational systems (or regulatory focuses). The promotion motivational system is activated by growth needs; therefore, it is characterized as the motivation to attain growth and nurturance. The promotion system concentrates on achievements and aspirations and endeavors to bring one's actual self into alignment with one's ideal self. The promotion system concerns positive states, the pleasurable presence of positive outcomes or the painful lack of positive outcomes (e.g., gains vs. non-gains). Promotion-focused individuals use approach strategic means to achieve their goals. Moreover, the promotion system is inclined toward challenges and risks (Higgins, 1997, 1998; Higgins et al., 2001; Florack et al., 2013). In contrast, the prevention motivational system is activated by safety needs, therefore, it is connected with the motivation to achieve security. The prevention system concentrates on commitments, duty, fulfillment of responsibilities, protection and safety, and bringing one's actual self into alignment with one's ought self. The prevention system is concerned with negative states and the avoidance of negative outcomes; therefore, it is focused on the pleasurable absence of negative outcomes and the painful presence of negative outcomes (e.g., non-losses vs. losses). Prevention-focused individuals are prone to use avoidance strategic means to achieve their goals. Furthermore, the prevention system is connected with a preference for stability (Higgins, 1997, 1998; Higgins et al., 2001; Florack et al., 2013). The aforementioned characteristics of motivation systems lead to the conclusion that a promotion- and prevention-focus may lead to different decisions and the use of different decision-making strategies.

Higgins (1997; 1998; Higgins et al., 2001) postulated, on the one hand, that promotion and prevention motivational systems can be understood as a relatively stable, chronic disposition but, on the other, momentary situations may temporarily induce either a promotion or prevention focus. The chronic disposition might have been developed from an early age during socialization (Higgins et al., 2001; Lockwood et al., 2002) while the situational motivation could have been influenced by the characteristics of a task (Shah et al., 1998; Zhou and Pham, 2004), and the experiences of the individual in a present or preceding context (Higgins et al., 1994). Therefore, motivational systems can be treated as a chronic individual difference variable that can also be measured using the Regulatory Focus Questionnaire (Higgins et al., 2001). It can also be situationally induced using different priming tools like feedback messages or task instructions with gain/non-gain information (activation promotion motivation) or non-loss/loss information (activating prevention motivation; Förster et al., 2003), or by a task in which the participants are asked to generate reports of their hopes/aspirations and duties/obligations (Chernev, 2004). There is lack of studies on decision making that analyze the role of situationally induced promotion and prevention motivation concurrently that would take the individual's chronic motivational system into account. However, it seems worthwhile investigating the mechanisms underlying decision making, taking the two aspects of motivational orientation into account. Therefore, in our studies we investigated the role of both chronic and situationally induced motivation.

### The promotion and prevention motivational focuses and people's financial decision making

During the last decades, many studies on the chronic motivational systems and situational motivation have shown that the promotion and prevention motivational focuses described by Higgins may influence people's decisions. Previous studies have shown that when multiple pieces of information are available, promotion motivated individuals process different kinds of information than prevention motivated people (Chernev, 2004; Florack et al., 2010). Promotion motivated individuals are focused on positive information, while prevention motivation leads to an attentiveness to negative information (Keller and Bless, 2008). Moreover, individuals with a promotional system are more likely to have inner impulses and emotions while making their choices (Pham and Avnet, 2004, 2009; Florack et al., 2010; Greifeneder and Keller, 2012). Förster et al. (2003) showed that people with chronic or situationally-induced promotion motivation perform faster and less accurately in simple drawing tasks compared to participants with prevention motivation.

Recent studies proffer that the promotion and prevention motivational systems exert an important impact on judgments, decisions, choices, and behavior in consumer contexts (Florack et al., 2010; Rybarczyk-Adamska et al., 2012). The results showed that people prefer those characteristics and product types that are compatible with their motivational system (Chernev, 2004; Foerster and Werth, 2007). Moreover, people would rather select those products that were advertised in accordance to their motivational system (Aaker and Lee, 2001; Cesario et al., 2004). A series of studies (Herzenstein et al., 2007) showed that promotion-motivated people declare a higher propensity to buying novel, high-tech goods than prevention-motivated people. What is more, promotion-focused people own more newly launched high-tech products than prevention-focused consumers. Wu and Kao's (2011) studies have shown that promotion-motivated individuals tend to select a greater variety of products than prevention-motivated individuals when they are allowed to buy only one item at a time for each consumption occasion (choice sequence). However, when consumers are allowed to buy several items at a time for each following consumption occasion, the effect is exactly the opposite—prevention-motivated people select a greater variety of products. Other studies showed that prevention-motivated people rate the utilitarian characteristics of products higher than promotion-motivated individuals, while people who have a promotion system rate hedonistic product characteristics higher (Chernev, 2004; Zawadzka and Niesiobędzka, 2010; Roy and Ng, 2012).

Far fewer studies have been conducted on the relations between motivational orientation and financial behavior. To the best of our knowledge, there are only two studies that have investigated the role of promotion/prevention motivational systems in the savings area. The studies of Cho et al. (2014) showed that in accordance with the Regulatory Focus Theory, the promotion motivational system is connected with positive attitudes toward saving. The Regulatory Focus Theory distinguishes between two types of goals: promotion goals associated with safety needs and prevention goals related to nurturance needs (Higgins, 1987). Cho et al. (2014) found evidence that both prevention-related and promotion-related saving goals increased the likelihood of intentional saving, which seems to be contrary to the theoretical framework. Moreover, participants who were promotion motivated were less prone to save for prevention goals, while for prevention-motivated individuals the effect was exactly the opposite.

There are only a few studies on motivational systems and investment decisions. Zhou and Pham (2004) postulated that investors identify and categorize financial instruments using separate mental accounts in which the financial product may be seen as representative of promotion vs. prevention. In their study, investments in stocks and trading accounts were related to promotion motivation, while mutual fund or Individual Retirement Account (IRA) investments were connected to prevention motivation. Moreover, Zhou and Pham's (2004) study showed that people who make choices regarding promotion investment products are more sensitive to gains, while people who make financial decisions regarding promotion instruments are more sensitive to losses. Promotion motivation is linked with approaching growth from the status quo, while prevention motivation is connected with avoiding the aggravation of a status quo, therefore, Florack et al. (2013) postulated that, consequently, prevention-motivated people are supposed to use less risky and more conservative tactics in pursuing a goal, while individuals with a promotion focus tend to even follow high-risk opportunities. Levine et al. (2000) conducted a study that showed that in the nonfinancial context of decisions (participants indicated whether or not they have seen a nonsense word presented earlier during the study), situationally promotion-motivated people make riskier decisions than situationally prevention-motivated individuals. There is also one study in the financial context that showed that prevention-motivated people tend to invest in financial instruments with minimal risk and small returns because those instruments are rated high on the crucial dimension of safety (Florack and Hartmann, 2007). However, four experiments (Scholer et al., 2010) provided evidence that individuals in a state of loss (perception of the negative shift from the status quo) with prevention motivation (both chronic and situationally-induced) exhibited risk seeking as long as the risky option was the only way to return to the previous status quo. However, if a sure (safe) option is available and it can lead back to the reference point (previous status quo), prevention motivation predicts risk aversion. These findings are in accordance with the theoretical framework, while Higgins (2009) stated that people who are prevention-motivated are more sensitive to negative shifts from the status quo than to the positive ones, while the effect for promotion-motivated individuals is exactly the opposite. Chernev's (2004) studies confirmed Higgins' statement and showed that prevention-motivated individuals have a greater preference for the maintenance of the status quo than promotion-motivated ones. Moreover, the results of these studies showed that framing the decision as either a gain or loss does not reverse the above mentioned preference for status quo maintenance.

The results described in the literature provide evidence that the motivational systems (described in the Focus Regulatory Theory) are influencing people's decision making however they are not giving any direct indicators as to how the motivational systems might influence financial decisions (also the risky once), especially when the individual is making decisions that are crucial for everyday life, for instance, whether to spend money for consumption immediately or postpone this decision and to later save or invest this amount. Therefore, in our studies we investigated how promotion and prevention motivational systems influence the propensity to consume, save, and invest. The distinction between saving and investing seems to be crucial. Both financial decisions are connected to delayed gratification, however, investing unlike saving is associated with the probability of losing money (the level of riskiness depends on the type of financial instrument) and, at the same time, investing is connected more with the possibility of changing the financial status quo. Therefore, the question arises as to whether a higher promotion orientation would motivate people to invest (as a way to multiply their money), while a higher prevention orientation would result in a greater propensity to save money (as a way of maintaining the financial status quo), or perhaps promotion motivation would lead to a preference for saving over consumption (collecting more money and meeting growth needs), and investing over saving (multiplying the money already owned), when prevention-motivated people would prefer both saving and investing over consumption whilst only taking into account relatively safe financial instruments (to feel financially secure). Moreover, to date there is only one study indicating that motivational systems influence people's financial risk preferences (the aforementioned study of Florack and Hartmann, 2007). Therefore, in order to verify and complement these results, in our study we not only analyze the propensity to invest but also the risk preferences in the investment and gambling domains, taking into account both chronic and situationally induced motivation.

## Present studies

Human choices and decisions are influenced by motivational systems described in the Focus Regulatory Theory. However, as mentioned previously, there is relatively little research involving the influence of motivational systems on people's financial decisions. There is a particular lack of in studies targeted at people's consumption, saving and investing preferences, and only one study on their financial risk preferences in the context of promotion and prevention motivation.

Therefore, the present studies aimed to examine the relationship between people's promotion and prevention motivation and their propensity to invest, undertake investment risks, and assume financial risks in gambling tasks in both the gain and loss decision-making frame. In our studies we investigated the role of two aspects of motivational orientation: (a) chronic motivational system—treated as a personality trait and relatively stable inclination; (b) situationally induced state—which can be induced by different external stimuli.

Based on the results of the studies mentioned earlier, the following questions were formulated: (1) Is the influence of motivational systems the same in terms of the propensity to invest and the tendency to make risky investment decisions? (Study 1 and Study 2), (2) Is the role of motivational systems in risk preferences the same across different financial domains (investing and gambling)? (Study 1 and Study 2), and: (3) Is the role of motivational systems in relation to risk preferences in gambling tasks the same when the decision is in a gain or loss frame, or will this role be different? (Study 1 and Study 2).

Moreover, we aimed to investigate how chronic motivational systems confronted with situationally induced promotion and prevention motivation would affect people's propensity to invest and embrace financial risks. The question which arose here concerns the interaction of both aspects of motivational orientation, namely, whether they will be additionally strengthened when chronic and situationally induced motivation is compatible. And, what is more important, what happens when they are incompatible—will financial decisions and choices be more driven by a chronic motivational system or a situationally induced one? (Study 2).

## Study 1—chronic motivational system and people's propensity to invest and take financial risks

The main goal of this study was to examine the relationship between people's chronic promotion and prevention motivational system and their propensity (1) to invest, and (2) to take financial risks in both the gain and loss decision-making frame.

The research tool that was used in the study also allowed us to investigate the relationship between people's chronic promotion and prevention motivational system and their propensity to consume and save.

### Methods

#### Participants

The first study was conducted on a Polish national representative sample, recruited on an on-line panel (CAWI). A total 1093 people participated in the study, with 558 women and 534 men aged 18–87 years (*M* = 36, *SD* = 12.84). The Ethics Board of the Institute of Social Science at the University of Warsaw approved the study, which was carried out in accordance with the Board's recommendations.

#### Study design and materials

The first study was a correlation study. The chronic motivational system (promotion and prevention) was measured using the Regulatory Focus Questionnaire (RFQ) by Higgins (Higgins et al., 2001). The RFQ consists of 11 questions, six of them are promotion scale items (e.g., compared to most people, are you typically unable to get what you want out of life?), five of them are prevention scale items (e.g., growing up, would you ever “cross the line” by doing things that your parents would not tolerate?). Participants had to answer the questions on a scale of 1–5 (never or seldom to very often—questions 1–8, never true—very often true—question 9, certainly false—certainly true—questions 10 and 11). The score of the promotion system is counted as the sum of the answers to the questions from the promotion scale. The score of the prevention system is counted analogously. Both RFQ scales exhibited good internal reliability (α = 0.73 for the Promotion scale; α = 0.080 for the Prevention scale; Higgins et al., 2001). The propensity to make financial choices was measured with one question tool in which the participants were asked to select one category of financial activity (consuming, saving, or investing) on which they would spend a hypothetical PLN 10,000 (as used in previous studies—Sekścińska, 2015a; Sekścińska et al., 2016). The tool also included information on the meaning of consuming, saving, and investing in the context of the study: consuming in the sense of spending money on products or services; saving meaning keeping the money in a nonprofitable (or almost nonprofitable) form without any risk of loss, for instance, a deposit in a noninterest-bearing bank account. Investing was defined as allocating funds to financial instruments that can generate profits but with the risk of losses, e.g., stocks or mutual funds.

The propensity to take financial risks in a gambling task in the gain and loss decision making frame was measured using two questions. In both questions, the participants were asked to choose between a sure (PLN 1000, equivalent to approximately $250) and a probable option (50% chance of PLN 0, 50% chance of PLN 2000, as well as PLN 2000, equivalent to approximately $540). Question 1 was in the gain frame, while question 2 was in the loss frame.

#### Procedure

At the beginning, the participants were asked to complete the RFQ questionnaire. They then answered the questions that measure the propensity to make financial choices and take financial risks in an order of rotation. At the end of the study, the participants filled in their metrical data (e.g., age, sex, and level of completed education).

### Results

A two-way analysis of variance (ANOVA) was conducted to explore the difference in the chronic promotion and prevention system between participants opting for different categories of financial activity (consumption, saving and investing). A significant effect was observed for both the promotion [*F*_(2, 1091)_ = 13.52, *p* < 0.001, η^2^ = 0.03, Table 1] and the prevention [*F*_(2, 1091)_ = 5.44, *p* < 0.005, η^2^ = 0.02, Table 1] systems. Bonferroni corrected *post-hoc* comparisons show that the differences in the levels of the promotion system between all three groups were significant (investing vs. saving: *p* < 0.005; saving vs. consumption: *p* < 0.002; investing vs. consumption: *p* < 0.001). Significant differences in the levels of the prevention system were observed between consumption and saving (*p* < 0.005), and between consumption and investing (*p* < 0.01).

**Table 1:** **Mean chronic promotion and prevention motivation depending on financial choice**.

	***M***	**95% Confidence interval**
**PROMOTION MOTIVATION**		
Consumption	19.00	[18.64, 19.37]
Saving	19.81	[19.60, 20.03]
Investing	20.51	[20.09, 20.94]
**PREVENTION MOTIVATION**		
Consumption	14.88	[14.38, 15.37]
Saving	15.75	[15.47, 16.04]
Investing	15.85	[15.37, 16.33]

To verify the differences in the promotion and prevention systems between participants who chose sure and probable options in a risk propensity (gambling) task (both in the gain and loss frame), four *t*-test analyses were conducted. In the gain frame, significant differences were observed in the level of the promotion system [*t*_(1091)_ = 2.886, *p* < 0.005, *Cohen's d* = 0.23, Table 2], while the difference in the level of the prevention system was not significant [*t*_(1091)_ = 1.261; *p* > 0.05; *Cohen's d* = 0.10, Table 2]. Analogously, in the loss frame, significant differences were observed in the level of the promotion system [*t*_(1091)_ = 2.026, *p* < 0.05, *Cohen's d* = 0.15, Table 2], while the difference in the level of the prevention system was not significant [*t*_(1091)_ = 1033; *p* > 0.05; *Cohen's d* = 0.07, Table 2].

**Table 2:** **Mean chronic promotion and prevention motivation depending on the decision-making frame and the preferred option (sure vs. probable)**.

	***M***	***SD***
**GAIN FRAME OF DECISION MAKING**		
**Promotion motivation**		
Sure option	19.69	2.88
Probable option	20.37	3.00
**Prevention motivation**		
Sure option	15.67	3.70
Probable option	15.29	3.65
**LOSS FRAME OF DECISION MAKING**		
**Promotion motivation**		
Sure option	19.47	2.95
Probable option	19.90	2.89
**Prevention motivation**		
Sure option	15.82	3.95
Probable option	15.55	3.62

The results of Study 1 showed that a higher level of chronic promotion motivation was associated with the preference for investments over savings and savings over consumption. Moreover, those participants that tended to invest or save money rather than consume money had a higher level of chronic prevention motivation. In terms of risky financial decisions in both the gain and loss decision frame, a higher level of chronic promotion motivation was related to the preference for the non-sure option over the sure one. However, there were no significant differences in the level of chronic prevention motivation between participants who chose the sure and non-sure options.

## Study 2—chronic motivational system vs. situationally induced promotion and prevention motivation and people's propensity to invest and take financial risks

The study here presented aimed to check whether and how situationally induced promotion and prevention motivation would affect people's propensity to invest and take financial risks in the context of their own chronic motivation. It was hypothesized that the effect of the activation of motivation systems may be different depending on the level of chronic promotion and prevention motivational systems.

The research tool used in the study also allowed us to investigate the relationship between people's chronic promotion and prevention motivational system and their propensity to consume and save.

### Method

#### Participants

The first study was conducted on a Polish national representative sample, recruited on an on-line panel (CAWI). Study 2 was conducted in two waves with a 2-week break between the first and second wave. A total of 1096 people took part in the first wave of the study; however, only 548 people took part in both the first and second wave of the study. Data was only analyzed from those people who completed both parts of the study. The final research group consisted of 332 women and 216 men aged 18 to 76 years (*M* = 43, *SD* = 14.79). The Ethics Board of the Institute of Social Science at the University of Warsaw approved the study, which was carried out in accordance with the Board's recommendations. At the end of the study, the participants were fully debriefed.

#### Experimental design and materials

The study was conducted with two experimental conditions. Participants were randomly assigned to each condition where the promotion or prevention systems were induced. The motivation system that was induced was the between-subjects IV. The second between-subjects IV was the level of the chronic promotion system (*chronic promotion*), and the third between-subjects IV was the level of the chronic prevention system (*chronic prevention*). The first within-subjects IV was the financial choice category measured on three levels: consumption, saving, and investing. People's propensity for different financial choices, indicated by the amount of money assigned to different categories by the participants was the first DV. The second within-subjects IV was the investment category measured on three levels: bonds, balanced mutual funds (that invest 50% in stock and 50% in bonds), and stocks. People's propensity to take an investment risk indicated by the amount of money assigned by the participants to different investment categories was the second DV. The propensity to take financial risks in the gain decision-making frame and the propensity to take financial risks in the loss decision-making frame were the third and fourth DV's that were indicated as a preference of the non-sure option over the sure one.

Situational motivation was activated using four sentences that the participants were supposed to read. There were two versions of the sentences. The first version referred to the four aspects of the promotion system (1. In life, it is important to strive to succeed; 2. It is always worth proceeding to accomplish your goal; 3. When you open your own business it is worth checking how much you can gain; 4. In life, you need to make risky decisions), and the second version referred to four aspects of the prevention system (1. In life, it is important to avoid potential failures; 2. It is always worth proceeding to avoid debacles; 3. When you open your own business it is worth checking how much you can lose; 4. In life, you need to make safe decisions), as described by Higgins (Higgins, 2009).

The sentences used to activate situational motivation were chosen based on the results of the pilot study. A total of 42 Polish adults took part in the on-line pilot study. They were randomly assigned to the promotion or the prevention condition. Participants read a set of four sentences and after pressing the “next screen” button, they were asked to recall the sentences they had just read and write the first three associations that came into their minds. It was accepted that good answers were comprised of responses where all three associations were related to the promotion or prevention pride, respectively, or when two associations were related to the promotion or prevention system and the third was neutral in terms of motivation regulation but linked with the words in the read sentences (e.g., business and life). Forty one participants' associations were consistent with our assumptions.

The chronic motivational systems (promotion and prevention) were measured analogously to study 1 using the Regulatory Focus Questionnaire (RFQ) by Higgins (Higgins et al., 2001).

To measure people's propensity for different financial choices, the participants were asked to distribute a hypothetical amount of PLN 10,000 (equivalent to approximately $2500) between consumption, savings, and investment. The tool also included information on what consuming, saving, and investing meant in the context of the study. The explanation was exactly the same as the explanation used in Study 1. Propensity to financial risk in a gambling task in the gain and loss decision-making frame was measured using two questions, in exactly the same way as in Study 1. Finally, the participants' propensity to take investment risks was assessed. All the participants were asked to create an investment portfolio by indicating what percentage of a hypothetical amount of money (PLN 10,000, which is equivalent to about $2500) they would want to allocate to a variety of financial market instruments. The participants could invest their hypothetical money in bonds, balanced mutual funds (investing 50% in stock and 50% in bonds), and stocks. The participants had the opportunity to select one or more of the instruments mentioned above (e.g., they could allocate the entire amount of money to stocks or divide it between two or more investment instrument categories). The objective of this task was to check the participants' propensity to invest in bonds, mutual funds, and shares (indicated by the percentage of the amount allocated to the relevant investment instruments). The task was also to check the general riskiness of the created portfolio (*riskiness of portfolio*). The indicator of the overall riskiness of the created portfolio reflected the percentage of shares (instruments that are affected by a significant risk of loss) in the portfolio (DV). The indicator was based on the following formula: 0 × percentage of bond + 0.5 × percentage of fund + 1 × percentage of shares (therefore, 0 was the lowest possible value of the indicator, meaning the safest portfolio, and 100 was the highest possible value of the indicator, signifying the riskiest portfolio).

#### Procedure

The second study was conducted in two waves with a 2-week break between the first and second wave. In the first part of study, the participants completed the Regulatory Focus Questionnaire (RFQ) and provided demographic information. Two-weeks later, we sent an invitation to the participants of the first study phase to take part in the second phase of the study. Those who decided to take part in the second wave were randomly assigned to the promotion or prevention experimental group. Firstly, the participants read the sentences that activated promotion or prevention regulatory motivation. They were then asked to write three things that they remember from the sentences they had just read (manipulation check). After that, they completed the task in order to measure their propensity for different financial choices, financial risks, and investment risks. The tasks were presented in random order. At the end of the study, the participants were fully debriefed.

### Results

To conduct the statistical analysis, the chronic promotion and chronic prevention variables were re-coded into binary variables (low vs. high level), the medians were split points.

A 2 (situational motivation) × 2 (chronic promotion level) × 2 (chronic prevention level) × 3 (financial choice category) mixed-design analysis of variance (ANOVA) was conducted. The main effect of situational motivation was not significant [*F*_(1, 539)_ = 0.12, *p* < 0.74, η^2^ = 0.001, Table 3]. No significant main effects of chronic promotion [*F*_(1, 539)_ = 1.47, *p* < 0.23, η^2^ = 0.003, Table 3] and chronic prevention [*F*_(1, 539)_ = 1.47, *p* < 0.23, η^2^ = 0.003, Table 3] were observed either. A significant main effect of the financial choice category was found [*F*_(1.68, 905.15)_ = 92.09, *p* < 0.001, η^2^ = 0.15, Table 3]. There were significant differences in the amount of money assigned between consumption and saving (*p* < 0.001), consumption and investing (*p* < 0.001), and saving and investing (*p* < 0.001).

**Table 3:** **Mean amounts of money (in PLN) assigned depending on the situational motivation, the chronic promotion level, the chronic prevention level and the financial choice category**.

	***M***	**95% Confidence interval**
**SITUATIONAL MOTIVATION**		
Promotion	3334,55	[3328.46, 3338.21]
Prevention	3334,55	[3329.48, 3339.63]
**CHRONIC PROMOTION LEVEL**		
Low	3331.78	[3326.76, 3336.79]
High	3336.11	[3331.18, 3341.04]
**CHRONIC PREVENTION LEVEL**		
Low	3331.78	[3327.29, 3336.27]
High	3336.11	[3330.70, 3341.52]
**FINANCIAL CHOICE CATEGORY**		
Consumption	2236.64	[2061.79, 2411.49]
Saving	4903.07	[4645.98, 5160.16]
Investing	2862.12	[2604.58, 3119.66]

The two-way interactions between the chronic promotion level and the financial choice category [*F*_(1.68, 905.15)_ = 2.04, *p* = 0.14, η^2^ = 0.004], and between the chronic prevention level and the financial choice category [*F*_(1.68, 905.15)_ = 0.65, *p* = 0.50, η^2^ = 0.001] were not significant. However, a significant interaction between the situational motivation and the financial choice category occurred [*F*_(1.68, 905.15)_ = 11.52, *p* < 0.001, η^2^ = 0.02].

The three-way interactions between the chronic promotion, the chronic prevention, and the financial choice category [*F*_(1.68, 905.15)_ = 2.56, *p* < 0.07, η^2^ = 0.01], as well as between the situational motivation, the chronic prevention, and the financial choice category [*F*_(1.68, 905.15)_ = 2.46, *p* < 0.08, η^2^ = 0.01] showed a tendency toward significance. No significant three-way interaction between the situational motivation, the chronic prevention, and the financial choice category was observed [*F*_(1.68, 905.15)_ = 0.25, *p* < 0.74, η^2^ < 0.01].

To correctly interpret the significant interaction between the situational motivation and the financial choice category pair-wise comparisons, using the Bonferroni correction for multiple comparisons were conducted. They showed that people whose promotion motivation was activated tended to spend significantly less money on consumption and saving and more money on investing than participants from the situational prevention group (consumption: *p* = 0.005; saving: *p* = 0.001; investing: *p* < 0.001, Table 4).

**Table 4:** **Mean amounts of money (in PLN) assigned depending on the situational motivation and the financial choice category**.

	**Consumption *M* (*SD)***	**Saving *M* (*SD)***	**Investing *M* (*SD)***
**SITUATIONAL MOTIVATION**			
Promotion	1999.27 (1912.60)	4421.53 (3127.08)	3579.20 (3302.05)
Prevention	2493.43 (2130.61)	5237.59 (2768.89)	2277.29 (2583.57)

This was followed by 2 (situational motivation) × 2 (chronic promotion level) × 2 (chronic prevention level) × 3 (investment category) mixed-design analysis of variance (ANOVA). The main effect of the situational motivation was not significant [*F*_(1, 539)_ = 1.03, *p* = 0.31, η^2^ = 0.002, Table 5]. No significant main effects of chronic promotion [*F*_(1, 539)_ = 1.03, *p* = 0.31, η^2^ = 0.002, Table 5] and chronic prevention [*F*_(1, 539)_ = 1.03, *p* = 0.31, η^2^ = 0.002, Table 5] were observed. A significant main effect of the investment category was found [*F*_(1.87, 1009.89)_ = 69.05, *p* < 0.001, η^2^ = 0.11, Table 5]. There were significant differences in the amount of money assigned between bonds and balanced mutual funds (*p* < 0.001), balanced mutual funds and stocks (*p* < 0.001), and bonds and stocks (*p* < 0.001).

**Table 5:** **Mean percentage of money assigned depending on the situational motivation, the chronic promotion, the chronic prevention, and the investment category**.

	***M***	**95% Confidence interval**
**SITUATIONAL MOTIVATION**		
Promotion	33.33	[33.32, 33.34]
Prevention	33.34	[33.33, 33.35]
**CHRONIC PROMOTION MOTIVATION**		
Low	33.33	[33.32, 33.34]
High	33.34	[33.31, 33.34]
**CHRONIC PREVENTION MOTIVATION**		
Low	33.34	[33.33, 33.35]
High	33.33	[33.32, 33.34]
**INVESTMENT CATEGORY**		
Bonds	47.76	[44.90, 50.62]
Balanced mutual funds	30.45	[28.11, 32.79]
Stocks	21.80	[19.37, 24.23]

The interaction between the investment category and the chronic promotion level was significant [*F*_(1.87, 1009.89)_ = 2.88, *p* < 0.05, η^2^ = 0.01], however, no significant interaction between the investment category and the chronic prevention level was observed [*F*_(1.87, 1009.89)_ = 1.10, *p* = 0.33 η^2^ = 0.002]. An interaction effect between the situational motivation and the investment category was also observed [*F*_(1.87, 1009.89)_ = 9.75; *p* < 0.001, η^2^ = 0.02].

The interaction effect between the chronic prevention level, the chronic promotion level, and the investment category was significant [*F*_(1.87, 1009.89)_ = 3.16, *p* < 0.05, η^2^ = 0.01]. However, the interactions between the situational motivation, the investment category, and the chronic promotion level [*F*_(1.87, 1009.89)_ = 0.40, *p* = 0.66, η^2^ < 0.01], as well as the chronic prevention level [*F*_(1.87, 1009.89)_ = 1.08, *p* = 0.34, η^2^ = 0.01] were not significant.

Finally, the interaction between the chronic promotion level, the chronic prevention level, the situational motivation and the investment category was not statistically significant [*F*_(1.87, 1009.89)_ = 0.08, *p* = 0.91, η^2^ < 0.01].

To correctly interpret the significant interactions between the situational motivation and the investment category, as well as between the chronic promotion level and the investment category the Bonferroni corrected *post-hoc* tests were conducted. The results showed that participants with a higher chronic promotional motivation were prone to spend significantly more money on balanced investment funds (*p* = 0.05) and less money on stocks (*p* = 0.001) compared to lower promotional people. No difference was observed between the high and low promotional groups in terms of their propensity to invest in bonds (*p* = 0.23). Moreover, Bonferroni corrected *post-hoc* comparisons revealed that participants with a situational promotional motivation were less prone to invest in bonds (*p* < 0.001) and demonstrated a greater tendency to invest in balanced investment funds (*p* < 0.002) than participants with situational prevention motivation. There was no significant difference in the propensity to invest in stocks between participants with situational promotion and prevention motivation (*p* = 0.16).

In order to perform follow-up tests, the data set was split according to the levels of variables involved in significant interaction between the chronic prevention level, the chronic promotion level, and the investment category. The set was first divided into the three investment category subsets (bonds, balanced investment funds, and stocks). Three 2 (chronic prevention level) by 2 (chronic promotion level) ANOVAs were conducted. The interaction between the chronic promotion and the prevention level was significant for the most risky category of investment instruments—stocks [*F*_(1, 543)_ = 5.69, *p* < 0.05, η^2^ = 0.01]. No significant interaction was observed for bonds [*F*_(1, 544)_ = 2.20, *p* = 0.14, η^2^ = 0.004] and balanced investment fund categories [*F*_(1, 544)_ = 0.50, *p* < 0.48, η^2^ = 0.001]. To correctly interpret the obtained interactions, the Bonferroni corrected *post-hoc* tests were run. The results showed that participants who were low-prevention motivated were prone to invest less money in stocks than those who were high-prevention motivated as long as the low-prevention motivated person was also low-promotion motivated (*p* < 0.02).

This was followed by a 2 (situational motivation) × 2 (chronic promotion level) × 2 (chronic prevention level) analysis of variance (ANOVA) to analyze the differences in the riskiness of the created portfolios. The main effect of situational motivation was significant [*F*_(1, 539)_ = 11.16, *p* < 0.001, η^2^ = 0.02, Table 6]. No significant principal effects of chronic promotion [*F*_(1, 539)_ = 2.87, *p* = 0.09, η^2^ = 0.005, Table 6] and chronic prevention [*F*_(1, 539)_ = 1.84, *p* = 0.18, η^2^ = 0.003, Table 6] were observed.

**Table 6:** **Riskiness of created portfolio depending on the situational motivation, the chronic promotion, and the prevention motivational system**.

	***M***	**95% Confidence interval**
**SITUATIONAL MOTIVATION**		
Promotion	41.08	[37.77, 44.38]
Prevention	32.97	[29.54, 36.41]
**CHRONIC PROMOTION MOTIVATION**		
Low	39.08	[35.68, 42.48]
High	34.97	[31.63, 38.31]
**CHRONIC PREVENTION MOTIVATION**		
Low	38.67	[35.62, 41.71]
High	35.38	[31.71, 39.05]

Furthermore, the interaction between chronic promotion and chronic prevention was significant [*F*_(1, 539)_ = 5.17, *p* < 0.05, η^2^ = 0.01], while no significant interaction effects between situational motivation and both chronic levels of motivational systems [chronic promotion: *F*_(1, 539)_ = 0.68, *p* = 0.41, η^2^ = 0.001; chronic prevention: *F*_(1, 539)_ = 1.60, *p* = 0.21, η^2^ = 0.003] were observed. The interaction effect between the chronic prevention level, the chronic promotion level and situational motivation was not significant [*F*_(1, 539)_ = 0.13, *p* = 0.71, η^2^ < 0.001].

To correctly interpret the obtained significant interaction between the chronic promotion and the chronic prevention motivational systems pair-wise comparisons, using the Bonferroni correction for multiple comparisons were run. The results showed that participants with a low chronic promotion system were prone to build significantly more risky portfolios than people with a high promotion system if their chronic prevention system was low (*p* < 0.005). No significant difference was observed between participants differing in terms of the chronic promotion system when their prevention system was high (*p* = 0.84).

To verify the differences in the promotion and the prevention systems and situationally induced motivation between participants who chose a sure or probable option in a risk propensity (gambling) task, two 2 (chronic promotion) × 2 (chronic prevention) × 2 (situational motivation) ANOVA's were conducted. One for the gain decision frame and one for the loss decision frame. At the beginning, we analyzed choices in a gain frame. The main effect of situational motivation was significant [*F*_(1, 540)_ = 5.78, *p* = 0.02, η^2^ = ‘0.01, Table 7] as was the main effect of the chronic promotion motivation system [*F*_(1, 540)_ = 10.25, *p* < 0.001, η^2^ = 0.02, Table 7]. No significant main effects of the chronic prevention motivational system was observed [*F*_(1, 540)_ = 2.81, *p* = 0.09, η^2^ = 0.005, Table 7]. The interaction between chronic promotion and chronic prevention was not significant [*F*_(1, 540)_ = 0.29, *p* < 0.54, η^2^ = 0.001]. The two-way interactions between situational motivation and chronic promotion [*F*_(1, 540)_ = 2.23, *p* < 0.14, η^2^ = 0.004], and chronic prevention [*F*_(1, 540)_ = 0.45, *p* < 0.51, η^2^ = 0.001] were also not significant. No significant three-way interaction between chronic promotion, chronic prevention, and situational motivation was observed [*F*_(1, 540)_ = 0.74, *p* < 0.39, η^2^ = 0.001].

**Table 7:** **Mean preferred option (sure vs. probable) depending on the chronic motivation systems and situational motivation on the gain and loss decision frame**.

	***M***	**95% Confidence interval**
**GAIN FRAME OF DECISION MAKING**		
**Situational motivation**		
Promotion	1.23	[1.18; 1.28]
Prevention	1.15	[1.09; 1.19]
**Chronic promotion motivation**		
Low	1.13	[1.08; 1.18]
High	1.24	[1.19; 1.29]
**Chronic prevention motivation**		
Low	1.22	[1.17; 1.26]
High	1.16	[1.11; 1.21]
**LOSS FRAME OF DECISION MAKING**		
**Situational motivation**		
Promotion	1.90	[1.86; 1.94]
Prevention	1.79	[1.74; 1.83]
**Chronic promotion motivation**		
Low	1.88	[1.84; 1.93]
High	1.80	[1.76; 1.85]
**Chronic prevention motivation**		
Low	1.86	[1.81; 1.90]
High	1.83	[1.78; 1.83]

The choices in a loss frame were then analyzed. The main effect of situational motivation was significant [*F*_(1, 540)_ = 13.33, *p* < 0.001, η^2^ = 0.03, Table 7], as was the main effect of the chronic promotion motivation system [*F*_(1, 540)_ = 6.35, *p* < 0.02, η^2^ = 0.01, Table 7]. No significant main effects of the chronic prevention motivational system were observed [*F*_(1, 540)_ = 1.18, *p* < 0.28, η^2^ = 0.002, Table 7]. The interactions between chronic promotion and chronic prevention [*F*_(1, 540)_ = 0.05, *p* = 0.82, η^2^ < 0.001], and between situational motivation and chronic prevention [*F*_(1, 540)_ = 0.19, *p* < 0.67, η^2^ < 0.001] were not significant. However, the interaction between situational motivation and chronic promotion [*F*_(1, 540)_ = 10.27, *p* < 0.001, η^2^ = 0.02] was significant. No significant three-way interaction between chronic promotion, chronic prevention, and situational motivation was observed [*F*_(1, 540)_ = 0.28, *p* < 0.60, η^2^ = 0.001]. In order to correctly interpret the obtained interaction effect, the data set was split according to the levels of the chronic promotion motivation system. Further χ^2^ tests showed that situational promotion induced participants to choose the not-sure option significantly more often than people from the prevention experimental group when their chronic promotion level was high [$\chi_{\left(1\right)}^{2}$ = 18.55, *p* < 0.001]. No significant difference was observed for people with low chronic promotion motivation [$\chi_{\left(1\right)}^{2}$ = 0.82, *p* = 0.85].

The results of Study 2 showed that people with induced promotion motivation are prone to spend less money on current consumption and savings and more on investments than the participants from the prevention experimental group.

Regarding investment instrument preferences, the participants with higher chronic promotion motivation were prone to spend more money on balanced funds and less money on stocks than low-promotion motivated individuals. Low-chronic prevention motivated people were less prone to invest in stocks than high-chronic prevention motivated individuals as long as their promotion motivation level was also low. Moreover, the participants with induced promotion motivation were prone to invest less in bonds and more in balanced funds than the prevention experimental group.

Significant differences were also observed in terms of the riskiness of the created portfolio. Those participants with their promotion system activated built more risky portfolios than individuals with their prevention system induced. Moreover, the chronic level of motivational systems was also important. Participants with a low level of their promotion system built more risky portfolios than high-promotion motivated individuals as long as their prevention system was also low.

## Discussion

The conducted research has shown, as was predicted that motivational systems (or regulatory focuses) can be related with financial behaviors, in it with the propensity to make risky financial choices. Based on the Regulatory Focus Theory (Higgins, 1998; Higgins et al., 2001), the promotion motivation system should be connected to an openness to more risky financial decisions and behaviors, while the prevention motivation system should be associated with the avoidance of risky behavior. Since investing can be connected with both relatively safe and aggressive financial instruments; therefore, investing can meet both the safety and the growth needs (e.g., depending on type of investment). The meeting of safety needs is crucial for people with high prevention motivation while the satisfaction of growth needs is very important for promotion-motivated individuals. Therefore, the relationship between the propensity to invest and the level of chronic promotion and prevention motivation is not so obvious.

Our studies showed the important role of the chronic motivation system on people's propensity to invest, but only when they can spend their money on one financial choice category (consume, save, or invest). When they can dispose their money between all categories, the role of chronic motivation is not significant; however, the role of situational motivation becomes important in such situations. Our results demonstrated that in the single choice task people who prefer to invest have a higher chronic promotion and prevention motivation than those who would rather spend their money. Moreover, people who prefer to invest have a higher level of chronic promotion yet the same level of chronic prevention than those who choose to save. When the decision maker can distribute the money between consumption, saving and investing, situationally induced promotion motivation results in a higher propensity to invest and a lower inclination to consume and save than induced prevention motivation, independently from the chronic level of the promotion and the prevention motivational systems. The willingness to meet safety needs (prevention motivation) and growth needs (promotion motivation) is the explanation for the preference to refrain from consuming any spare money among those with high promotion and in prevention-focused people. People with a high prevention focus want to secure their financial future, which is why they probably only take safe investments into account. From this point of view, the difference between saving and investing is not so significant, while the level of chronic prevention among people who decide to save their money is similar to the level of prevention among those who prefer to invest. However, high promotion-motivated people are focused on growth; hence, they may only take those investment instruments into account that could bring large returns. This can be an explanation for why they prefer investments over savings. The lack of evidence for the role of motivational systems in financial preferences when the decision maker can divide her/his money between different financial categories is surprising. Further studies are needed to confirm this result and to find its explanation.

The results of our studies have also confirmed the role of the motivational focuses in explaining people's investment risk preferences. Situationally promotion-motivated people tend to build more aggressive investment portfolios than situationally prevention-motivated individuals. Our findings are in agreement with the theoretical framework (Higgins, 1997, 1998; Florack et al., 2013) and the results from the previous study on non-financial risk and situational motivation (Levine et al., 2000), and show that also in the context of financial decisions the induction of promotion motivation results in a higher risk propensity than the induction of prevention motivation.

Our studies also showed that people with situationally-induced promotion motivation avoid bonds compared to situationally prevention-motivated individuals, which is not surprising as long as promotion motivation is related to approaching growth from the status quo, while prevention motivation is connected with avoiding the aggravation of a status quo, which is in accordance with the findings from the Florack and Hartmann (2007) study. Moreover, the level of interest in stock investments is similar for both promotion and prevention situationally motivated people. Promotion motivated individuals prefer balanced mutual funds to stocks.

Our studies also demonstrated that a higher level of chronic promotion motivation seems to be associated with the tendency to spend more money on balanced investment funds and less money on bonds. These results may be surprising, especially in the context of previous studies. Zhou and Pham (2004) postulated that investors identify and categorize financial instruments using separate mental accounts in which the financial product may be seen as representative of promotion (e.g., stocks) vs. prevention (e.g., mutual funds). The results of other studies that were conducted in the consumer decision context showed that people prefer those characteristics and product types that are compatible with their motivational system (Chernev, 2004; Foerster and Werth, 2007). Therefore, it could be expected that people will behave analogously to their consumer choices in investment decisions. The question arose as to why both chronic- and situationally-induced promotion motivation results in a balanced investment fund preference (and not in a stock preference). Promotion motivated people are more prone to take risks but they are also focused on meeting growth needs (Higgins, 1997, 1998). Investing all their money in stocks is very risky and may bring high returns as well as large losses of money. Serious money loss (which can happen in the case of investments in stocks alone) may make it difficult to meet growth needs but a very small profit (in the case of investments exclusively in bonds) approaches this goal only marginally. Balanced funds seem to be the most attractive for promotion-motivated people albeit they are more risky than bonds, they may bring higher returns than bonds, but are less risky than investments in stocks alone. In terms of risky investment choices, chronic prevention motivation is related to the propensity to invest in stocks but only if the level of an individual's chronic promotion motivation is low. It seems as though chronic promotion motivation plays a much more important role in explaining people's investment choices than chronic prevention motivation.

Our studies also provided the evidence that chronic and situational motivation may explain people's risk preferences in gambling tasks. The results showed that people who prefer non-sure options are stronger chronic promotion motivated that those who choose the sure option in both the gain and loss decision frame. Moreover, situationally promotion motivated people decide to choose non-sure options more often than situationally prevention motivated people in both the gain and loss decision frame. However, the relationship in the loss frame is significant only when the decision-maker is high chronic promotion motivated.

The obtained results are in accordance with the theoretical assumptions (Higgins, 1997, 1998).

However, the expectation that decisions made under conditions of risk in gambling tasks would be different depending on the frame of the decision failed to confirmed. This expectation was based on earlier findings from studies on risk preferences (Kahneman and Tversky, 1979, 2000; Kusev et al., 2009; Vlaev et al., 2010; Kusev and van Schaik, 2011). The results of our studies failed to support this hypothesis. Nevertheless, our findings are in accordance with the results of Chernev's (2004) studies, which showed that framing the decision as either a gain or a loss does not reverse promotion and prevention motivated people's preferences for status quo maintenance.

The studies conducted by us on risk-taking were both in the investment and gambling financial decision domain. The role of chronic and situational motivation seems to be similar across the domains but the two tools that were used in the studies cannot be compared, particularly because there was no middle option in the gambling task that would be less risky and potentially less profitable than the non-sure option but more potentially profitable than the sure option. It could be expected that promotion motivated people would choose the middle option in a gambling task, as they did in the case of investment tasks. Further, studies are needed to verify whether the relationship between promotion and prevention motivation and risk preferences is stable across different financial domains.

The vast majority of previous research on the Regulatory Focus Theory (Higgins, 1998; Higgins et al., 2001) investigated the role of the chronic motivational system or the effects of activation of promotion vs. prevention motivation; however, not many studies have been conducted on both the influence of the chronic and activated motivational system at the same time. In our second study, both chronic and situational promotion and prevention motivation were included. The results showed that the chronic motivational system and situationally induced motivation seem to work rather independently (the only significant interaction effect was observed in the gambling task in a loss frame of decision). It would be worthwhile to investigate this finding further.

## Author contributions

KS, DM, and AT planned the research and designed the studies. KS analyzed the data. KS and DM wrote the manuscript along with inputs from AT during revisions.

## Funding

The current project and the publication were financed by the resources of Polish National Science Centre-NCN (Award number: DEC-2013/11/B/HS6/01163).

### Conflict of interest statement

The authors declare that the research was conducted in the absence of any commercial or financial relationships that could be construed as a potential conflict of interest. The reviewers TZ, AC and the handling Editor declared their shared affiliation, and the handling Editor states that the process nevertheless met the standards of a fair and objective review.
